# Treating tobacco dependence: guidance for primary care on life-saving interventions. Position statement of the IPCRG

**DOI:** 10.1038/s41533-017-0039-5

**Published:** 2017-06-09

**Authors:** O. C. P. Van Schayck, S. Williams, V. Barchilon, N. Baxter, M. Jawad, P. A. Katsaounou, B. J. Kirenga, C. Panaitescu, I. G. Tsiligianni, N. Zwar, A. Ostrem

**Affiliations:** 1 Department of Family Medicine, CAPHRI, Maastricht University, Maastricht, The Netherlands; 2 International Primary Care Respiratory Group, Aberdeen, UK; 3 Andalusian Health Service (SAS), Tobacco group of GRAP (Primary Care Respiratory Group), Andalusia, Spain; 4 Southwark Clinical Commissioning Group, London, UK; 5 Faculty of Medicine, School of Public Health, Imperial College London, London, UK; 6 Pulmonary Medicine, Medical School, National and Kapodistran University of Athens, Evaggelismos Hospital, Athens, Greece; 7 Lung Institute and Division of Pulmonary Medicine, Makerere University College of Health Sciences, Kampala, Uganda; 8 Family Medicine Solo Practice, RespiRo- Romanian Primary Care Respiratory Group, Bucharest, Romania; 9 Clinic of Social and Family Medicine, Faculty of Medicine, University of Crete, Crete, Greece; 10 School of Public Health and Community Medicine, UNSW Australia, Sydney, NSW Australia; 11 General Practitioner, Gransdalen Legesenter, Oslo, Norway

## Abstract

Tobacco smoking is the world’s leading cause of premature death and disability. Global targets to reduce premature deaths by 25% by 2025 will require a substantial increase in the number of smokers making a quit attempt, and a significant improvement in the success rates of those attempts in low, middle and high income countries. In many countries the only place where the majority of smokers can access support to quit is primary care. There is strong evidence of cost-effective interventions in primary care yet many opportunities to put these into practice are missed. This paper revises the approach proposed by the International Primary Care Respiratory Group published in 2008 in this journal to reflect important new evidence and the global variation in primary-care experience and knowledge of smoking cessation. Specific for primary care, that advocates for a holistic, bio-psycho-social approach to most problems, the starting point is to approach tobacco dependence as an eminently treatable condition. We offer a hierarchy of interventions depending on time and available resources. We present an equitable approach to behavioural and drug interventions. This includes an update to the evidence on behaviour change, gender difference, comparative information on numbers needed to treat, drug safety and availability of drugs, including the relatively cheap drug cytisine, and a summary of new approaches such as harm reduction. This paper also extends the guidance on special populations such as people with long-term conditions including tuberculosis, human immunodeficiency virus, cardiovascular disease and respiratory disease, pregnant women, children and adolescents, and people with serious mental illness. We use expert clinical opinion where the research evidence is insufficient or inconclusive. The paper describes trends in the use of waterpipes and cannabis smoking and offers guidance to primary-care clinicians on what to do faced with uncertain evidence. Throughout, it recognises that clinical decisions should be tailored to the individual’s circumstances and attitudes and be influenced by the availability and affordability of drugs and specialist services. Finally it argues that the role of the International Primary Care Respiratory Group is to improve the confidence as well as the competence of primary care and, therefore, makes recommendations about clinical education and evaluation. We also advocate for an update to the WHO Model List of Essential Medicines to optimise each primary-care intervention. This International Primary Care Respiratory Group statement has been endorsed by the Member Organisations of World Organization of Family Doctors Europe.

## Background: the tobacco epidemic

This paper provides an update to the original paper IPCRG Consensus statement: Tackling the smoking epidemic—practical guidance for primary care of 2008 published in this journal.^1^ Tobacco dependence is a deadly epidemic killing up to half its users.^2^ There are currently one billion smokers in the world and 80% live in low and middle income countries.^3^ Tobacco use is the leading cause of premature death globally due to the many diseases, which are attributed wholly or partially to smoking resulting in nearly six million deaths a year.^4, 5^ This annual total is higher than the combined mortality from malaria, tuberculosis and human immunodeficiency virus (HIV).^6^ Based on current trends, tobacco is expected to be responsible for 10% of global deaths, or eight million deaths a year by 2030, 80% in low and middle income countries: this includes a 9% decline in high income, but a doubling in low and middle income to 6.8 million.^7^ This is creating growing health inequalities at national and global levels due to the strong association of tobacco use with socio-economic deprivation.

There is a global commitment to tackle the epidemic. In 2011, the UN General Assembly adopted a political declaration that committed member states to the prevention and control of non-communicable diseases (NCDs). This included a target of a 25% reduction in premature mortality from NCDs by 2025 (“25 × 25”) and targets for nine risk factors, including a 30% relative reduction in smoking prevalence.^8^ This is anticipated to bring most health and economic benefit to low and middle income countries and included modelling of smoking attributable diseases such as cardiovascular diseases (CVDs), chronic respiratory diseases, tuberculosis, cancer, diabetes and respiratory infection.^9^ However, the latest modelling suggests a more ambitious target of a 50% reduction in smoking prevalence relative to 2010 levels would be the most feasible way to ensure the 25 × 25 target is reached.^9^

Tobacco control policies have already been very successful, lending force to the UN’s specialised agency, the World Health Organisation’s (WHO), Framework Convention on Tobacco Control (FCTC).^10, 11^ However, while public health measures such as raising taxes on cigarettes, plain packaging and advertising controls will prevent many from taking up smoking and encourage some to quit, many of those who are most tobacco dependent will not be able to quit without treatment from a health-care professional. Therefore, the FCTC includes a specific article on treating tobacco dependence, Article 14, which argues that cessation support is essential, and will also have a synergistic effect on the other control measures. The WHO has also called specifically for smoking cessation to be integrated into primary health care, as it is seen as the most suitable health setting for providing advice and support on smoking cessation globally. Yet only 15% of the world’s population has access to appropriate cessation support.^9, 12^

This statement is about treatment and not about prevention. However, we recognise that primary care has a role to play in prevention in measures such as:

- public advocacy for the rigorous uptake of FCTC wherever the opportunity arises;
- acting as role models by not smoking, maintaining smokefree premises, and placing no smoking posters in prominent places in their offices;
- application of family medicine principles to identify and coach other members of the family who may be at risk of taking up smoking if there is a family member who smokes;
- recording smoking status so that it is possible to monitor the impact of the interventions.

However, with regard to the FCTC, the most important duty is article 14—to identify smokers and treat them, with whatever resources are available, taking note of new research findings.

Individuals gain significant health benefits by quitting smoking (Table 1).^13^ Helping smokers quit is one of the most cost-effective interventions available to clinicians.^14^ Where smokers are aware of the dangers, most want to quit.^1, 2^ However, in countries such as China, that consumes 30% of the world’s tobacco, there is less awareness of the dangers and many less affluent people who remain more dependent smokers do not yet have intention to quit.^15^ Nicotine is addictive; tobacco dependence is recognised by WHO as a chronic relapsing condition^16–18^ that normally begins in childhood or adolescence.^19, 20^ Success rates are low for unaided attempts to quit.^21^ Clinicians should apply the same principles to tobacco dependence as to other chronic relapsing conditions. This includes offering patients accurate and timely diagnosis, evidence-based treatment that aims to co-produce outcomes with the patient using behavioural and pharmacological support, and regular review, anticipating the likelihood of relapse and, therefore, offering a system for repeated support to quit.

**Table 1:** Health benefits of smoking cessations

Time since quitting	Beneficial health changes that take place
24 h	Lungs start to clear out mucus and other smoking debris
48 h	Carbon monoxide will be eliminated from the body. Ability to taste and smell is greatly improved
72 h	Breathing becomes easier. Bronchial tubes begin to relax and energy levels increase
2–12 weeks	Circulation improves
3–9 months	Coughs, wheezing and breathing problems improve as lung function is increased by up to 10%
1 year	Risk of a heart attack falls to about half that of a smoker
10 years	Risk of lung cancer falls to half that of a smoker
15 years	Risk of heart attack falls to the same as someone who has never smoked

From: McEwen, A., McRobbie, H., West, R. and Hajek, P. (2006) Manual for Smoking

Cessation: a guide for counsellors and practitioners. Oxford: Blackwell

## Introduction

The health benefits of smoking cessation, and the efficacy and cost-effectiveness of medical treatment for tobacco dependence, are well established.^22^

For pharmacological therapies, it has been estimated that one person will successfully quit (achieve 6-month abstinence) for every 6–23 people treated.^23^ Given that approximately half of all long-term smokers will die of a smoking-related illness,^24^ smoking cessation is a highly effective preventive strategy, compared with other widely used primary prevention interventions, such as the use of statins, and, therefore, deserves to be made a priority by clinicians and policy-makers. This comparison can be demonstrated by numbers needed to treat (NNT) to achieve an intervention effect (Table 2). When it comes to treatment of tobacco dependence, the NNT to achieve one long-term quitter is low. Absolute quit rates data can differ between trials due to a number of variables, e.g., the definition of cessation, the socio-economic status of the population included and level of follow-up.^22^ To obtain one long-term quitter by using brief advice (<5 min) the NNT is 40,^23^ although the NNT can be as low as 10 when medication is combined with behavioural support.^24^ The NNT to achieve abstinence can be translated into NNT to prevent premature death if we take into account that for every two smokers who stops long-term, one would otherwise die from a smoking-related disease.^1, 24^ This figure is very low compared to other interventions such as the use of statins to prevent death (NNT 107), the primary prevention of MI or stroke death over one year (NNT 700)^5, 25^ or the 10-year prevention for cervical cancer screening (NNT 1140)^26^ (see Table 2).

**Table 2:** Comparison of number needed to treat (*NNT*) to prevent one death. Smoking cessation medication is usually used for 3–6 months, while statins or antihypertensive medication might be used throughout life

NNT comparison		
Intervention	Outcome	NNT
Smoking cessation behavioural support plus		
- NRT	Long-term quitter/premature death	23/46
- Bupropion	Long-term quitter/premature death	18/36
- Varenicline	Long-term quitter /premature death	10/20
Statins as primary prevention	Prevent one death over 5 years	107
Antihypertensive treatment for mild hypertension	Prevent one stroke or MI death over 1 year	700
Screening for cervical cancer	Prevent one death over 10 years	1140

Health professionals such as family doctors working in primary care can play a vital role in helping their patients quit smoking. In 2008, the International Primary Care Respiratory Group (IPCRG) published the first Consensus Statement on practical smoking cessation guidance for primary care.^1^ The IPCRG is working closely with the WHO on the prevention and treatment of chronic lung disease as part of its NCD programme and it is important we offer practical guidance to support the WHO’s stated ambition for the primary-care diagnosis and treatment of tobacco dependence (http://www.who.int/tobacco/publications/building_capacity/training_package/treatingtobaccodependence/en/). A recent review indicates that brief advice to stop from a health-care worker, automated text messaging, and cytisine are globally affordable interventions.^27^ The next step is to review the essential medicines list produced by the WHO Model List of Essential Medicines^28^ and to increase the smoking cessation products that are included.

## Opportunities for primary care

Primary health-care encounters represent frequent and important opportunities to identify tobacco use, provide advice and help people to quit.^29, 30^ The majority of smokers with a family physician see them at least once a year.^31, 32^ Many people consider their family physician as a key influence and source of advice about smoking.^33, 34^ However, despite the opportunity, and the effectiveness of interventions to help patients quit, many smokers do not receive support.^35^ There are many stated barriers to intervene including time constraints, confidence in raising the issue, competence, lack of training and resources, health professionals’ beliefs that brief advice is unlikely to be effective or that patients are not motivated to quit, and reluctance to jeopardise the doctor–patient relationship by giving unwelcome advice_._^36–42^

In reality, however, patient satisfaction with the consultation is generally higher when smoking is addressed.^43, 44^ There are many short and long-term beneficial health changes to motivate patients to quit (Table 1).

In addition health-care professionals’ own smoking behaviour and their attitude towards smokers and their motivation to help people quit is important, particularly in relation to the most dependent people from low socio-economic backgrounds.^44^

Since tobacco smoking is addictive, causes substantial morbidity and mortality and as a result causes huge costs for the health-care system, we believe it is our clinical responsibility to use every opportunity to prompt and help smokers to quit in an evidence-based way.

## Guidance

Primary-care health professionals have an important role to play in triggering quit attempts and assisting with those quit attempts. Effective smoking cessation interventions in primary care are based on an awareness of which strategies have been shown to work (Table 3), and on making the most of available resources.

**Table 3:** Adapting Very Brief Advice on Smoking to your context

Intervention	Rationale	Considerations
Establish smoking status (ASK): “Do you smoke or use a waterpipe?”	Knowledge of smoking status is a prerequisite to any intervention	How will smoking status be recorded and how will you ensure that smoking status is recorded for all patients?
Advise that the best thing that the patient can do for their current and future health is to stop smoking (ADVISE) “Smoking tobacco leads to lung disease, cancers, heart disease and an early death. Stopping smoking is the single most important thing that you can do to improve your health.”	In some countries, knowledge about the harmful effects of smoking is high and so there is no need to advise smokers that smoking is harmful. Additionally, a meta-analysis revealed that offering advice without the offer of support did not prompt quit attempts. Where cessation services (face-to-face or quitlines) you can simply advise on the best way of quitting: “The best way of quitting is with a combination of behavioural support and medication. We have a local, friendly stop smoking service who are experts in this and I can refer you if you’d like?”	Does this element need to include more information on the harmful effects of smoking and the benefits of cessation? Are resources needed to supplement the advice? The advice will be dependent upon what support is available
Act on patients' response to advice (ACT) by either: (a) facilitating referral to cessation services, or alternative support (e.g., prescribing or referring to a pharmacy or doctor with appropriate recommendations); (b) making a note in their medical records that advice has been delivered if they do not want to quit	Behavioural support (from a trained specialist practitioner or primary care professional) is the most effective method of supporting a quit attempt, but quit lines or self-help materials can be used. Recording that advice has been delivered is a prompt to health professionals that they need to deliver it again at the next appropriate contact with the patient	If referral for support is not available, can support be offered within your practice? What medications are available? Are written support materials or other resources (e.g., websites) available?

Key recommendations are summarised in Table 4.

**Table 4:** Key recommendations

Recommendation	Grade^a^
Make your practice ‘‘smoke free’’ by banning smoking on the premises, displaying information on smoking cessation in the waiting room, asking every patient about smoking status, and promoting smoking cessation services	B
Opportunistically provide brief, clear advice to quit whenever appropriate (doctors) and offer available assistance with any quit attempt	A
Train practice nurses and other staff to encourage smokers to quit and offer assistance	C
Recommend a local telephone counselling service (‘‘quit line’’), where available, to all smokers who indicate interest in quitting	A
Consider prescribing drug treatment for tobacco dependence (e.g., nicotine replacement therapy, bupropion, varenicline) to people who smoke 10 or more cigarettes per day, after consideration of contraindications and comorbidity	A
Tailor your approach to smoking cessation advice or treatment to the individual’s degree of readiness to quit	D
Use a non-judgemental communication style	C
Use motivational interviewing techniques^b^ to help people understand their own attitudes to smoking and quitting, make their own decisions and solve problems encountered during a quit attempt	B
Provide or arrange intensive behavioural counselling, where resources permit	A

^a^ Recommendations graded according to the Scottish Intercollegiate Guidelines Network system (described at http://www.bmj.com/cgi/content/full/323/7308/334 accessed January 2008)

^b^ Effective when provided by trained counsellors

### Use every opportunity to promote smoke-free living

Consider whether your practice can implement some or all of these strategies to raise awareness of the benefits of quitting and offer support.^45^ Become a ‘‘no-smoking’’ practice; institute a smoking ban on practice premises for staff as well as visitors. Place posters and smoking cessation literature in the waiting area. Set up systems to prompt you to ask each patient about smoking; routinely record smoking status in medical records, and review it at least yearly. A combination of these strategies, promoting use of effective medication and use of support services (such as telephone counselling services: ‘‘quit lines’’) can more than double quit rates among your patients, compared with no intervention.^45–47^

**Fig. 1:**
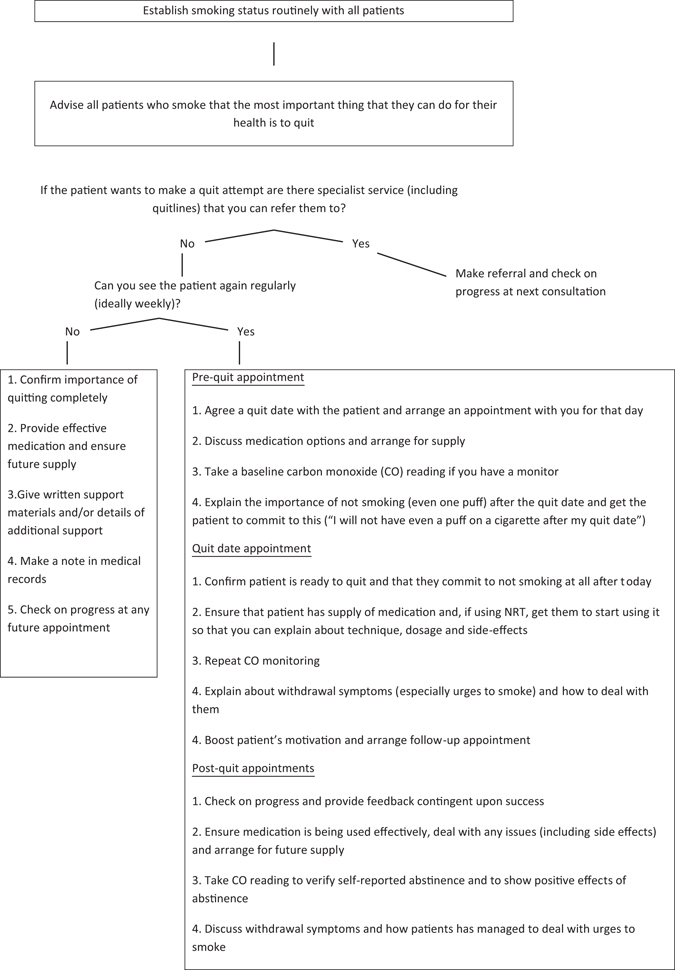
Deciding what smoking cessation interventions you can deliver

Opportunistically provide clear, personalised, non-judgemental advice to quit, and offer appropriate and available help to quit. For every 100 people who receive brief advice from a primary-care doctor to quit smoking, up to three extra people will succeed in quitting for at least 6 months than if no advice is given.^7, 9^ Approximately 40% of smokers make some attempt to quit after such advice.^7^ This shows that intervention really can make a difference (Fig. 1). We support the every contact count model (https://www.nice.org.uk/sharedlearning/making-every-contact-count-implementing-nice-behaviour-change-guidance), which requires every health-care professional in the system in contact with a smoker to have the competence and confidence to be able to help someone quit. More people see primary-care providers in a year, than any other health-care professional, and, therefore, primary care needs to get it right. Where people with respiratory conditions are managed in secondary care, smoking cessation should be the first line treatment for all smokers. Where primary care is the main caregiver, but refers to secondary care for an episode of care, for example from surgeons, anaesthetists and midwives then collaboration is essential as outcomes in surgery and maternity will improve with smoking cessation.

### Support smokers who want to quit

Whenever smokers indicate they would like to quit, offer your support.^45, 48–54^ Provide reliable verbal and written information and offer consultations to help them to plan the quit attempt. Refer people to quit lines, where available, as this will be of additional benefit. Services that actively call people back for follow-up counselling are more effective than those providing only counselling on demand.^46–51^

Prescribe drug treatment for tobacco dependence as indicated (Table 5). Pharmacotherapy, particularly when accompanied by behavioural support, significantly improves long-term quit rates compared with no treatment or placebo.^52^

**Table 5:** Pharmacotherapy for nicotine dependence

Medication^a^:
Any patient smoking more than 10 cigarettes a day or who smoke within 30 -60 minutes of waking will suffer from withdrawal symptoms and should be offered pharmacological support once they set a quit date. Remember to offer psychological support during the first 3 months of the cessation attempt
Nicotine replacement therapy (*NRT*)
NRT should not be combined with smoking. Its main effect is to reduce abstinence and help the patient through the first couple of months of craving. Most patients use too low doses for too short a time. They should use a dose that takes away abstinence symptoms. Most people need a full dose for 2–3 months, then they might gradually reduce the use over some months. Added success has been shown if NRT is started 14 days prior to quit date
Dosage: It is often wise to combine two different NRTs—a patch to cover most of the day and gum or other types of NRT (e.g. spray) for craving situations during daytime
Patch: Comes in 14 mg/24 h or 10 mg/16 h for light smokers or in 21 mg/24 h—15 mg/16 h for more heavy smokers. Some patients need more than one patch a day to keep the symptoms low
Side effects: Skin rash, allergy, insomnia, wild dreams
Gum, inhalers, lozenges, sublingual tablets: To be administered every 1–2 h for relief of symptoms while awake. Since nicotine is absorbed through the mucosa in the mouth it is important to instruct the patient in the use of gum carefully. Chew a few times on the gum then “park” it in the mouth
Side effects: local-sore dry mouth, dyspepsia, nausea, headache, jaw ache. Often dose dependent
Contraindication: Pregnancy (in some countries)
Varenicline (©Champix, ©Chantix)
Varenicline is a nicotinic receptor partial agonist. In addition to blocking the receptor it also stimulates it thus reducing abstinence. It is the first drug designed for smoking cessation. Results are promising with quit rates up to 44% in some studies
Dosage: Start 1 week before quit date: 0.5 mg for 3 days, 0.5 mg bid for 4 days, then 1 mg bid from quit date for 12 weeks
Side effects: nausea and headache. There is no danger of seizures. Risk of psychiatric side effects is the same as for other smoking cessation medications
Contraindication: Pregnancy
Bupropion (©Zyban)
Bupropion is the first medication proven to reduce the craving
Dosage: twice daily starting with one tablet a day for a week two weeks prior to quit date, then regularly 150 mg bid from quit date for 7–12 weeks
Adverse effects: insomnia, headache, dry mouth, dizziness, anxiety
Contraindications: Seizures, pregnancy, major depression, schizophrenia, drugs for treating depression or schizophrenia
Other medication:
Other drugs have shown to be effective in smoking cessation but are not licenced for use. The cost of these drugs are often low and should be considered in case cost is a limiting factor
Nortryptilyn (©Noritren) is cheap and has also been shown to be effective, but adverse effects that include sedation, dry mouth, lightheadedness and risks of cardiac arrhythmia in patients with CHD limit its application. It should thus be a second line agent
Cystisine (©Tabex) has a mechanism of action like Varenicline, binding to the nicotinic receptor. It has been used for smoking cessation in eastern European countries and has received increasing interest due to its low cost. Side effects include stomach-ache, dry mouth, dyspepsia and nausea
Which drug to advice?
Previous experience, availability, cost and patient’s preference will often guide the choice of medication. In a cost-effectiveness study from a high-income country, both NRT, Varenicline and Bupropion were shown to be cost-effective compared to placebo^b^. Varenicline was show to be most cost-effective

^a^ Adapted from: http://www.theipcrg.org/display/TreatP/Helping+patients+quit+smoking%3A+Desktop+Helper+No.+4+-+2nd+Edition

^b^ Hagen G., Wisløff T., Klemp M. Cost-effectiveness of varenicline, bupropion and nicotine replacement therapy for smoking cessation. Rapport fra Kunnskapssenteret nr. 10—2010. ISBN 978-82-8121-341-8 ISSN 1890-1298

Pharmacotherapy should be offered to people who smoke 10 or more cigarettes per day^53–55^ or who smoke within 30–60 min of waking, after consideration of contraindications and comorbidity. Selection of pharmacotherapy is based on clinical suitability, availability and patient choice.^55^

A motivational, non-judgemental style during consultations is more likely to engage patients.^56–59^ Take the role of an interested, empathising partner who asks questions that explore the smoker’s determination and ability to quit. Motivational interviewing acknowledges that the patient, not the doctor, is responsible for changing behaviour. Expired air carbon monoxide monitoring can validate self-reported quits but also act as a strong motivational tool.^60^ Nowadays carbon monoxide monitoring is not only used for research purposes, but also for clinical and this kind of feedback may considerably facilitate smoking cessation in primary care.^61^

### Make the most of available time—Very Brief Advice on Smoking (VBA)

The aim of smoking cessation interventions is first of all to trigger or prompt quit attempts, and then to assist with a quit attempt. There is a role for every primary health-care professional in prompting a quit attempt by delivering VBA. This involves establishing smoking status (ASK); advising on the benefits of cessation and/or making the offer of help to quit (ADVISE); acting on the patients response and making a referral/providing support to quit as appropriate (ACT) [Table 3]. VBA is a short evidence-based and effective intervention that does not require assessment of readiness to quit (as the US 5 A’s approach does) and which combines the Assist and Arrange elements.^62^ The key elements are providing VBA opportunistically to all patients and offering whatever assistance is available to help prompt and support quit attempts. If a general practitioner diagnosed someone with hypertension, would he or she wait until they were ready to be treated, or start to talk to them about a treatment plan ?

Effective strategies can be incorporated into routine primary-care encounters, even where available consultation time is limited (Fig. 1). In addition, before health workers even mention smoking, a practice can already have sent patients a strong visual “quit smoking” message through posters and literature in the waiting room^63^ (Table 4).

“Asking” should be accompanied by an appropriate entry in the medical notes: tobacco dependency should be part of past medical history: consider it a “vital sign.”^64^

### Harm reduction

For people who do not want, or who feel unable, to stop smoking, it could be beneficial to try to reduce harm from continued tobacco use. Options include reducing the amount of tobacco used; cutting down only or prior to stopping smoking (“cutting down to quit”) and smoking reduction and temporary abstinence from smoking.^65^ There is some evidence that smokers who regularly engage in temporary abstinence with the use of NRT are more likely to stop smoking in the future and there is evidence to support use of nicotine replacement prior to smoking cessation.^66^ Smokers who use NRT for smoking reduction are approximately twice as likely to progress to quitting than those who do not.^67, 68^

### Electronic cigarettes

There is considerable debate about the place of electronic cigarettes (ECs) in smoking cessation and harm reduction and, as yet, only a limited number of good quality studies, especially on the subject of cessation. There are indications that suggest that on average e-cigarettes improve success rates, but evidence is still preliminary.^69^ The debate illustrates the importance of acknowledging and understanding the context for implementing all scientific evidence and primary-care practitioners should be aware of and be open to changes in both the context and the evidence. The context includes different national or regional public health approaches towards harm reduction,^70^ interpretation of current evidence by different medical associations, legal frameworks and licensing decisions. Most governments have not licensed e-cigarettes for smoking cessation. In some countries, the medicinal products listed in Table 5 are hard to access or unaffordable, but e-cigarettes are available.

Based on the concentrations of chemicals in e-cigarettes, vapour would be expected to be less harmful than smoking, though not free of risk.^70^ As the evidence accumulates recommendations may change but at present it is recommended that patients be advised to use methods of quitting with strong evidence for effectiveness where these are available. If patients ask about e-cigarettes, they should be informed that there is currently little hard evidence on the effectiveness of e-cigarettes in smoking cessation. They should also be told that while they are likely to be substantially safer than smoking, they probably do carry some risk and long-term use should be avoided if possible. On the basis of current evidence, they should also be told that use of an e-cigarette may impede their chances of quitting smoking at a later date. E-cigarettes should never be advised as a life-style choice. The family doctor has to realise that he is not only treating the adult smoker but also the smokers’ family and for adolescents observing the use of e-cigarettes by parents may be a gateway to ultimately smoking.

## Groups that needs special attention

### Pregnant women

Smoking cessation interventions are particularly important during pregnancy as tobacco smoking in pregnancy remains one of the few preventable factors associated with stillbirth, complications in pregnancy, low birthweight, preterm birth^71, 72^ and has serious long-term health implications for women and infants including the development of asthma.^73^ Post-partum follow-up reduces relapse rates.

While smoking in pregnancy is decreasing in high-income countries, worldwide the prevalence of tobacco smoking and smokeless tobacco use among women is increasing and the WHO characterises this rise of tobacco use in young women in low-income, high-population countries as one of the most ominous developments of the tobacco epidemic.^74^ The use of carbon monoxide screening or monitoring can be an effective non-judgemental way of identifying maternal exposure to tobacco smoke that might not otherwise be discussed.^75, 76^

Nicotine replacement therapy may be appropriate, subject to local prescribing regulations, given that the dose of nicotine is lower than the dose from cigarettes. Single dose NRT will typically give less than half the amount of nicotine delivered via smoking, but without carbon monoxide and other toxins.^77^ An observational study has suggested combination NRT may be more effective than single form NRT (patch or rapid acting form) in pregnant women.^78^ This finding may be related to faster metabolism of nicotine in pregnancy.^67^ Using NRT is clearly better than smoking cigarettes and, therefore, most national smoking cessation guidelines weigh up the benefits as stronger than the risks and, therefore, recommend using some form of NRT during pregnancy. If used, oral forms are usually preferred due to lower total dose of nicotine. Bupropion and varenicline are not recommended in pregnancy.^79^ Breastfeeding mothers can use NRT since nicotine levels in the infant from NRT while breastfeeding are low and are unlikely to be harmful. Advise that they use it after breastfeeding, and if they relapse, similarly advise them to smoke immediately after the feed to allow more time for the toxins to leave the breast milk before the next feed.^80–82^

### Children and adolescents

Approximately 80% of smokers begin smoking during their teenage years. Nicotine dependence develops very rapidly in teenagers. Among teenagers, 10% do so within 2 days of inhaling from a cigarette for the first time, and 50% by the time they are smoking seven cigarettes per month.^83^

In general, teenagers care more about the immediate benefits to their appearance, current well-being and financial status than about future health gains. Therefore, it is useful to emphasise the following benefits of quitting in addition to long-term health: better physical appearance including teeth, avoiding bad breath, cost savings (e.g., calculate the amount spent per year on cigarettes) and better sexual performance.

### Sociocultural background and socioeconomic status

Cultural issues will differ between primary-care settings, depending on the country and its cultural norms, the sociocultural backgrounds of health professionals, and the socioeconomic status of their patients. Independent of desire to quit, groups who are socioeconomically deprived face additional challenges and may need more support to quit and avoid relapse. In Europe, for instance, quit rates seen over the past 10 years have been higher and uptake lower among groups with higher socioeconomic and education status.^84^ Those groups that have not yet benefited from the trend towards a smoke-free lifestyle stand to benefit most from well-directed support from their primary-care health professionals. Governments should take a firm stand in this regard, not being led by considerations of losing tax revenues by reduced sales of cigarettes.

## Patients with chronic diseases

It is important to target this large population of smokers for smoking cessation support given the role that smoking plays in causing or exacerbating many long-term conditions. Patients who continue to smoke despite suffering from a smoking-related condition are likely to be more dependent and thus require more support.

### Respiratory diseases

Smoking cessation is the key intervention for slowing progression of chronic obstructive pulmonary disease (COPD). There is a clear relationship between continued smoking and progression of COPD.^85^ Smoking in patients with COPD is associated with a faster decline in lung function and an increase in symptoms, as well as an increased risk for respiratory tract infection and hospitalisation^86^ and they show higher levels of depression and cigarette dependence.^87^ In people with asthma, smoking further impairs lung function, increases symptoms and exacerbations and impairs the effectiveness of treatment and quality of life.^88^ Successful cessation support strategies that are specific to patients with COPD include the use of pharmacotherapy, explaining the relationship between smoking and COPD and using spirometric results, CO monitors and “lung age” to increase motivation to quit.^89^

### Cardiovascular diseases

For patients with CVD smoking cessation is a key intervention.^90^ In people with coronary heart disease, smoking cessation is associated with a 36% reduction in mortality compared to those who continue to smoke. Many people admitted to hospital with CVD attempt to quit but it has been shown that follow-up in the community is important for sustained cessation.^91^

### Cancers

Continued smoking after a diagnosis of lung cancer is associated with worse quality of life and all clinical outcomes, including survival. People with successfully treated cancers who continue to smoke are at increased risk of a second cancer. Many studies report that smoking cessation after a diagnosis of lung cancer is associated with health improvements. There is good evidence that interventions in this group are effective and health-care personel must remember to offer both medication and psychosocial support for these patients.^92^

### Human immunodeficiency virus

Evidence suggests the prevalence of smoking among HIV infected people is higher than in the general population. Smoking in HIV infected people is associated with an increase in the occurrence of several communicable and NCDs. CVD risk is increased twofold and all-cause mortality is higher in HIV infected smokers compared to those who do not smoke. In one study, the risk of bacterial pneumonia decreased by 27% following smoking cessation.^93^ Smokers with HIV who receive and take antiretroviral therapy now lose more life years to smoking than they do to HIV itself.^94, 95^ The majority of smokers living with HIV report being interested in quitting, and a significant proportion have made recent quit attempts. Based on this evidence, every HIV care programme should include a service to screen for tobacco dependence and support smoking cessation for clients.

### Tuberculosis

Evidence exists that smoking greatly increases the risk of tuberculosis (TB).^96^ Surveys have shown that the prevalence of smoking among confirmed and presumptive TB patients is higher than in the general population.^97, 98^ Continued smoking during TB treatment is also associated with worse TB treatment outcomes (treatment failure and death).^99, 100^ There is good evidence that smoking cessation works for TB patients: abstinence rates greater than 50% can be achieved at the end of TB treatment with simple interventions such as brief advice.^101–103^

### Patients living with mental illness

Smoking is common among people diagnosed with psychiatric disorders and smoking rates are substantially higher than the general population.^104^ Depression is especially related to smoking.^105^ Patients with major mental illness die prematurely compared with people with non-major mental illness diagnoses. The causes are mostly due to an increase of smoking related diseases including heart disease, cancer, cerebrovascular, respiratory, and lung diseases.^106^

Therefore, smoking cessation is essential for people with mental illness. This goal is difficult because many health professionals are reluctant to tackle the problem, as they are afraid that quitting may worsen patients’ mental health. The fact is that smoking cessation actually improves mental health^107^ and should be recommended for all people with mental illness. Although mood problems are clearly different from psychotic disorders there are no indications that these patients should be treated differently in smoking cessation. Importantly, people with serious mental illness are almost as likely as the general population to want to stop smoking.^108^ There is also good evidence of efficacy of treatment in this group of patients.^109^ People with mental illness should be offered the same pharmacotherapy as the general population, although with closer monitoring^110^ of possible interaction between cessation medication and antipsychotic medication (for more information on drug interactions with tobacco smoke, please have a look at the fact sheet: Drug Interactions With Tobacco Smoke https://smokingcessationleadership.ucsf.edu/sites/smokingcessationleadership.ucsf.edu/files/Drug-Interactions-with-Tobacco-Smoke.pdf).

The doses of antipsychotic medication may need to be adjusted after smoking cessation.^111^

In meta analyses of varenicline, the most effective treatment in smoking cessation,^112^ there was no evidence of increased risk of suicide or attempted suicide, suicidal ideation, depression or death in varenicline users compared with placebo users.^113^ A recent large study examining safety and efficacy of varenicline, bupropion and nicotine patch in smokers with and without psychiatric disorders found no increase in neuropsychiatric adverse effects attributable to these medicines compared to placebo.^114^

In conclusion, smokers with chronic mental illness can quit smoking with minimal effects on psychiatric symptoms using standard cessation approaches, but with closer monitoring from experienced health professionals, and may need more quit attempts.^115, 116^ Their benefits in terms of years of life gained are more significant than the general population given their current premature mortality from cardio-respiratory conditions largely due to smoking.

### People using waterpipes—shisha

Waterpipe tobacco smoking (also known as shisha, hookah, or narghile smoking) is now a common form of tobacco use among adolescents worldwide, especially in the Middle East and Eastern Europe,^117^ and prevalence is rising in adults.^118^

Due to the mistaken perception of reduced harm associated with waterpipe tobacco use,^119^ asking specifically about waterpipe tobacco use in a consultation may uncover a significant tobacco history that would otherwise be missed.^120^ A recent Cochrane review concluded that waterpipe cessation interventions are sparse.^121^ However, given a shared nicotine and adverse health outcome component, interventions for cigarette cessation could be a useful consideration for waterpipe tobacco users.^121^

## People using cannabis and tobacco—joints

Fifty percent of cannabis users also smoke tobacco and this impairs successful withdrawal from cannabis.^122^ Tobacco smoking precedes cannabis use but the reverse gateway effect has also been described.^123^ Quitting tobacco can help people withdraw from other substance misuse so there appears to be a case for added value by helping people and designing services that assist with dual use. It has been suggested that the absence of an ICD code for cannabis withdrawal disorder may impact on the availability of pathways and services for this group. It, therefore, may fall to the general practitioner to support the cannabis and tobacco user in a quit attempt utilising what is available locally for each problem separately.

While there is limited evidence for what constitutes effective treatment, there is increasing concern about the impact of smoking cannabis and tobacco in ‘‘joints’’ and its impact on respiratory health.^124^ Studies suggest that simultaneously quitting both tobacco and cannabis may yield benefits at both the psychological and neurobiological level.^125^ One study involved a twelve-week program comprising computer-assisted delivery of Motivational Enhancement Therapy, Cognitive Behavioral Therapy, and Contingency Management, i.e., abstinence-based incentives for cannabis use disorder (CUD).^122^ In addition, participants were encouraged to complete an optional tobacco intervention consisting of nicotine-replacement therapy and computer-assisted delivery of a behavioural treatment tailored for tobacco and cannabis users. Simultaneously targeting tobacco during treatment for CUD did not negatively impact cannabis outcomes. Participation in the tobacco intervention was high, but cessation outcomes were poor suggesting that alternative strategies might be needed to more effectively prompt quit attempts and enhance quit rates. In terms of pharmacotherapy, a Cochrane systematic review has not found evidence for drugs that are used in other addiction therapy such as bupropion but that cannabinoid replacement therapy studies suggest a potential hope.^126^ Currently, however, it appears the best available evidence supports a tobacco intervention with pharmacotherapy in combination with a dual behavioural intervention.

### Need for gender-specific interventions

Our knowledge on gender shows that women need special attention as they are more predisposed to suffer the adverse consequences of tobacco smoking and need gender-specific counselling when quitting. Women develop COPD at an earlier age and with a greater degree of lung function impairment for a given amount of tobacco exposure.^127^ Although the benefit is clear, women have more difficulties in stopping smoking^128, 129^ and among quit attempters, women had 31% lower odds of successfully quitting.^130^

Another important factor that should be taken in consideration is the menstrual phase. Better treatment outcomes can be achieved by scheduling quit dates to coincide with the follicular phase of the menstrual cycle in female smokers.^131^ Nicotine and cotinine metabolism is faster in women than in men and is faster in women taking oral contraceptives compared with those who are not;^132^ there may be a need for higher doses of NRT in women. Regarding pharmacological approaches, NRT may have less beneficial effects.^133, 134^ Long-term maintenance of NRT treatment gains decrease more rapidly for women than men.^125^ By contrast, both buproprion, and varenicline^135^ are equally effective for men and women.

## Conclusions

There are growing health inequalities at national and global levels, with a strong association between tobacco use and socio-economic deprivation. For example, in the UK it is estimated one million people could be raised out of poverty if they quit smoking.^136^ Global targets to reduce premature deaths by 25% by 2025 require a substantial increase in smokers making a quit attempt especially in low and middle income countries. Physicians (including GPs) readily prescribe statins or antihypertensive treatment while the efficacy to prevent death is much better with smoking cessation treatment.

We strongly advocate for all primary-care professionals to avoid assumptions and to Ask (and record), Advise and Act. Act as if smoking—tobacco dependence—is a chronic relapsing-remitting disorder with highly effective and cost effective treatments, deliverable in primary care.

All can make an effective contribution in encouraging and helping existing adult smokers to quit and encouraging teenagers not to begin smoking. Evidence-based smoking cessation strategies can be incorporated into any primary-care practice, tailored to practice style, the patient demographics and the time and resources available.

Accordingly, the IPCRG guidance is offered as a set of suggested strategies based on those that have proved effective in their original sociocultural settings. In the absence of supportive government policies, implementation of the guidance will be particularly challenging for individual primary-care health professionals.
