# Erratum: Treating tobacco dependence: guidance for primary care on life-saving interventions. Position statement of the IPCRG

**DOI:** 10.1038/s41533-017-0048-4

**Published:** 2017-09-05

**Authors:** O. C. P. Van Schayck, S. Williams, V. Barchilon, N. Baxter, M. Jawad, P. A. Katsaounou, B. J. Kirenga, C. Panaitescu, I. G. Tsiligianni, N. Zwar, A. Ostrem

**Affiliations:** 1 Department of Family Medicine, CAPHRI, Maastricht University, Maastricht, The Netherlands; 2 International Primary Care Respiratory Group, Aberdeen, UK; 3 Andalusian Health Service (SAS), Tobacco group of GRAP (Primary Care Respiratory Group), Andalusia, Spain; 4 Southwark Clinical Commissioning Group, London, UK; 5 Faculty of Medicine, School of Public Health, Imperial College London, London, UK; 6 Pulmonary Medicine, Medical School, National and Kapodistran University of Athens, Evaggelismos Hospital, Athens, Greece; 7 Lung Institute and Division of Pulmonary Medicine, Makerere University College of Health Sciences, Kampala, Uganda; 8 Family Medicine Solo Practice, RespiRo- Romanian Primary Care Respiratory Group, Bucharest, Romania; 9 Clinic of Social and Family Medicine, Faculty of Medicine, University of Crete, Crete, Greece; 10 School of Public Health and Community Medicine, UNSW Australia, Sydney, NSW Australia; 11 General Practitioner, Gransdalen Legesenter, Oslo, Norway

**Erratum to**: *npj Primary Care Respiratory Medicine* (2017); doi:10.1038/s41533-017-0039-5; Published 09 June 2017

The original version of this article contained an error in the spelling of the author I. G. Tsiligianni, which was incorrectly given as K. W. I. G. Tsiligianni. This has now been corrected in both the PDF and HTML versions of the article.

